# The Photodegradation of Lignin Methoxyl C Promotes Fungal Decomposition of Lignin Aromatic C Measured with ^13^C-CPMAS NMR

**DOI:** 10.3390/jof8090900

**Published:** 2022-08-24

**Authors:** Bei Yao, Xiaoyi Zeng, Lu Pang, Xiangshi Kong, Kai Tian, Yanli Ji, Shucun Sun, Xingjun Tian

**Affiliations:** 1 School of Life Sciences, Nanjing University, Nanjing 210023, China; 2 Co-Innovation Center for Sustainable Forestry in Southern China, Nanjing Forestry University, Nanjing 210037, China

**Keywords:** photodegradation, litter, UV, lignin, fungal decomposition, ^13^C-CPMAS NMR

## Abstract

Solar radiation has been regarded as a driver of litter decomposition in arid and semiarid ecosystems. Photodegradation of litter organic carbon (C) depends on chemical composition and water availability. However, the chemical changes in organic C that respond to solar radiation interacting with water pulses remain unknown. To explain changes in the chemical components of litter organic C exposed to UV-B, UV-A, and photosynthetically active radiation (PAR) mediated by water pulses, we measured the chemistry of marcescent *Lindera glauca* leaf litter by solid-state ^13^C cross-polarization magic angle spinning (CPMAS) nuclear magnetic resonance (NMR) over 494 days of litter decomposition with a microcosm experiment. Abiotic and biotic factors regulated litter decomposition via three pathways: first, photochemical mineralization of lignin methoxyl C rather than aromatic C exposed to UV radiation; second, the biological oxidation and leaching of cellulose O-alkyl C exposed to PAR and UV radiation interacts with water pulses; and third, the photopriming effect of UV radiation on lignin aromatic C rather than cellulose O-alkyl C under the interaction between radiation and water pulses. The robust decomposition index that explained the changes in the mass loss was the ratio of aromatic C to O-alkyl C (AR/OA) under radiation, but the ratio of hydrophobic to hydrophilic C (hydrophobicity), the carbohydrate C to methoxyl C ratio (CC/MC), and the alkyl C to O-alkyl C ratio (A/OA) under radiation were mediated by water pulses. Moreover, the photopriming effect and water availability promoted the potential activities of peroxidase and phenol oxidase associated with lignin degradation secreted by fungi. Our results suggest that direct photodegradation of lignin methoxyl C increases microbial accessibility to lignin aromatic C. Photo-oxidized compounds might be an additional C pool to regulate the stability of the soil C pool derived from plant litter by degrading lignin methoxyl and aromatic C.

## 1. Introduction

Litter decomposition maintains C balance and nutrient cycling by altering the rate of C-based gaseous products returning to the atmosphere in terrestrial ecosystems [1,2,3]. However, the traditional exponential model based on soil biochemical cycle underestimates litter decomposition rates in arid and semiarid ecosystems, which has led researchers to consider the role of solar radiation in litter decomposition [4,5,6]. Photodegradation has been reported to positively affect the decay rates of litter decomposition in arid and semiarid ecosystems [7,8,9,10,11]. Recently, photodegradation can also be a significant driver of litter decomposition, such as tropical evergreen rainforests [12], subtropical evergreen broadleaved forests [13], temperate deciduous forests [14,15], and boreal forests [16,17,18]. In photodegradation, the radiation wavebands that have active effects extend from UV radiation to a visible region [16,19,20,21]. Different wavebands quantified with a weighting function showed different influences in magnitude and direction on litter decomposition [22].

The effects of solar radiation on litter decomposition depend on abiotic factors, such as precipitation and litter quality [23,24,25,26,27]. Leaching of soluble compounds caused by water pulses under frequent precipitation can lead to losses of dissolved organic matter and cellulose [23]. Dry-wet cycling in arid and semiarid ecosystems modulated by rainfall frequency can lead to the rupture of cell structures and fragmentation of leaf litter [24,25]. In addition, initial litter chemistry (litter C: N and lignin: N ratios, or lignin and polyphenolic compound contents) is related to photodegradative losses [5,15,28]. Photodegradation is also regulated by microorganisms in humid ecosystems [12]. In the process of microbial decomposition, bacteria colonize plant litter due to a large amount of labile carbon of plant litter at the primary stage of litter decomposition. However, at the late stage of litter decomposition, fungi can secrete oxidase to decompose a high proportion of recalcitrant C in litter [29,30]. The role of fungi in litter decomposition is quite clear. Yet, the effect of photodegradation on microbial decomposition is poorly understood.

Photodegradation leads to the breakdown of organic matter and affects litter C pools and fluxes through several decomposition pathways [5]. Photodegradation can directly photo-oxidize lignin (photosensitive compound) from organic C to CO_2_, CO, and CH_4_ [5,31,32]. Studies on the characterization of litter organic carbon by proximate analysis show that lignin concentration decreased in the process of photodegradation [19,28,31,32,33]. However, some studies using proximate analysis do not detect a reduction in lignin content in plant litter exposed to photodegradation [8,9,34]. An alternative chemical mechanism of photodegradation is that organic compounds are indirectly photo-oxidized by transferring electrons from photosensitive compounds to nonphotosensitive compounds [5,35]. Litter hemicellulose [8,36] and holocellulose (hemicellulose and cellulose) decrease under exposure to UV radiation [23] in semiarid grasslands by indirect photo-oxidization mechanism. Both direct and indirect photolysis are processes of photodegradation of organic C to inorganic C, which is also considered as a direct effect of photodegradation on litter decomposition [5,37]. In addition to direct photodegradation, photodegradation can affect subsequent microbial decomposition, which is called the photopriming effect [20,38,39]. The photopriming effect on litter chemistry exposed to UV radiation stimulates subsequent microbial decomposition by producing labile photodegraded material, and it is helpful for the efficiency of extracellular enzymes produced by microbial communities [19,20,34].

Early studies on photodegradation focused on the effect of UV radiation on litter decomposition [8,23], especially in UV-B radiation [32,34], until some studies extended the active effects of radiation wavebands on litter decomposition from UV radiation to visible regions [19,20,21,28]. Some studies show that UV-A radiation and blue light accelerate litter C loss [15,16]. Recent research suggests that blue light is the major driver in the process of photodegradation in terrestrial ecosystems by synthesizing 30 field experiments associated with spectral attenuation of solar radiation [40]. In addition, the relative importance of different wavebands to CO_2_ emission in solar radiation is quantified through a spectral weighting function [22].

Abundant papers refer to mass loss from litter materials with respect to decomposition indexes from proximate analyses (cellulose, hemicellulose, and lignin) [38]. However, few studies have analyzed changes in chemical compounds at the molecular level due to the structural complexity and heterogeneity of litter organic matter [39]. Moreover, if the initial lignin concentration of plant litter is relatively low, it is difficult to detect the UV radiation effect on lignin degradation using proximate analyses [23]. Thus, the methods of chemical assays for elucidating the chemical mechanism and degradation pathway of photodegradation need to be improved and verified in future research [8,35]. It has been established in recent years that nondestructive spectroscopic analytical methods, such as solid-state nuclear magnetic resonance (NMR) spectroscopy, are better choices than destructive approaches [39]. During microbial decomposition, ratios of alkyl C to O-alkyl C (A/OA) and carbohydrate C to methoxyl C (CC/MC) are the most robust indexes for predicting decay rates by solid-state NMR [41,42,43]. However, it is not clear which decomposition indexes can more accurately predict the changes in organic C during photodegradation.

There is no consistent conclusion about which chemical components account for the mass loss of plant litter exposed to solar radiation of different wavebands in ecosystems with complex environmental factors. Thus, we exposed plant litter to different radiation wavebands and water pulses and utilized solid-state ^13^C cross-polarization magic angle spinning (CPMAS) nuclear magnetic resonance (NMR) at a high resolution to efficiently observe the chemical changes of C components in the process of photodegradation. We hypothesized that (1) organic C loss caused by the interactive effects of radiation and water pulses would vary with the characteristics of chemical components; (2) photodegradation of organic C would exert a priming effect on subsequent organic C decomposition mediated by water pulses; and (3) the ratios of alkyl C to O-alkyl C (A/OA) and carbohydrate C to methoxyl C (CC/MC) would be the robust decay indicators of the decomposition rates under exposure to radiation and water pulses. Hence, understanding the decomposition pathways of organic C is helpful for improving the prediction power of decomposition models and interpreting how litter chemistry responds to climate change.

## 2. Materials and Methods

### 2.1. Experimental Materials

Marcescent leaf litter of *Lindera glauca*, a deciduous broadleaf tree, was collected in November 2015 during the start of leaves falling from a subtropical forest in the Zijin Mountains, Nanjing, China (32°50′ N, 118°48′ E). The forest features a northern subtropical humid monsoon climate, which is nearly snow-free during winter. The mean annual precipitation is 1106.5 mm, falling mostly from June to July, and the mean annual temperature is 15.4 °C. Based on the surface solar radiation dataset, the annual mean daily solar radiation in Nanjing is 146.48 W/m^−2^ [44,45]. Leaves of *L. glauca* fall in April of the following year when plants germinate new leaves. The ecological consequence of keeping dead biomass aboveground is not well understood, and studies have not reached an agreement on the ecological significance of marcescence [46]. However, *L. glauca* leaf litter with marcescent characteristics is an ideal material for studying abiotic decomposition mechanisms, while avoiding contact with soil microorganisms in humid subtropical ecosystems. Meanwhile, the vegetation is dominated by deciduous broad-leaved and evergreen coniferous species. Although *L. glauca* grows in the shadows of *Quercus variabilis* and *Pinus massoniana*, deciduous broad-leaved species commonly fall in autumn resulting in marcescent *L. glauca* receiving a large amount of solar radiation. Leaf litter was brought back to the laboratory, air-dried until a constant weight, and stored in a dark cabinet for five months at room temperature.

### 2.2. Experimental Design

We conducted a microcosm experiment to quantify the decomposition patterns of organic C (Figure S1). Two iron shelves (L × W × H: 1.20 m × 1.20 m × 1.60 m) with four layers were built. We installed five UV-B lamps on the second layer, five UV-A lamps on the third layer and five PAR lamps on the fourth layer, while there were no lamps on the first layer. Leaf litter of *L. glauca* (2 g air-dried mass) was placed in a PVC ring (50 mm high × 100 mm diameter) whose bottom consisted of a 0.50 mm nylon mesh. The bottom of each PVC ring was completely filled with leaf litter to ensure that litter received the same area of radiation. The PVC ring was placed on a tray (height 10 mm × diameter 110 mm) and set 30 cm below the lamps.

Four kinds of radiation wavebands were applied: a control (no radiation, 0 μW cm^−2^) identified as a blank (no radiation and no water) treatment to eliminate the potential activities of microbes, UV-B (103.54 μW cm^−2^), UV-A (296.00 μW cm^−2^), and PAR radiation (1355.44 μW cm^−2^). UV-B, UV-A, and PAR exposure were regulated by using a fluorescent UV-B (UVB-313, 280~320 nm), fluorescent UV-A (UVA-340, 320~400 nm), and white fluorescent lamp (PAR, 400~700 nm), respectively. A UV radiometer equipped with UV-B and UV-A probes was used to measure the spectral output of UV-B and UV-A radiation, and a photosynthetically active radiometer was used to measure the spectral output of PAR radiation (Photoelectric Instrument Factory of Beijing Normal University, Beijing, China). The lamps were operated from 8 a.m. to 6 p.m. every day to simulate solar radiation hours under natural conditions. The photodegradation effects of different radiation wavebands on mass loss and organic C were identified as the increase in mass loss and decrease of the relative abundance of organic C compared to the blank treatment.

To evaluate the interaction among photodegradation, biodegradation, and water-leaching, we set up two kinds of water treatments (R, radiation; R + W, radiation and water pulse). Alternatively, the photopriming effect was defined as the decrease in the relative abundance of organic C due to exposure to UV-B, UV-A, and PAR radiation compared to water treatment alone. The PVC rings containing leaf litter on another iron shelf were exposed to radiation and water pulses (R + W) to determine the interactive role of UV-B, UV-A, and PAR radiation and water pulses in litter decomposition. We used a syringe to add 30 mL distilled water into each PVC ring weekly. The amount of water (0.1 mL cm^−2^) is equivalent to 1 mm of rainfall (Gaxiola and Armesto, 2015). The experimental temperature was 25 °C. We harvested the samples after 75, 164, 288, 380, and 494 days. The collected litter samples were weighed to determine mass loss before being ground into powder. Litter was subsampled for extracellular enzyme assays. The remainder of the litter was oven-dried before being prepared for solid-state ^13^C-CPMAS NMR analysis of litter chemistry.

### 2.3. ^13^C CPMAS NMR Spectroscopy 

Litter C components were characterized by solid-state ^13^C-CPMAS NMR under the same conditions. Litter samples were analyzed at four recycling times during the stages of litter decomposition (0, 75, 288, and 494 days, Figure S2). The measurement was carried out by a Bruker Avance III 400 spectrometer (Bruker BioSpin Corporation, Switzerland) operating at a frequency of 100.6 MHz. Litter samples were packed into a 4-mm cylindrical zirconium rotor with Kel-F end-caps and run in a double resonance probe. NMR spectra were spun at a frequency of 10 kHz and recorded with a recycle time of 1 s, an acquisition time of 34 ms, and 2000 scans. To circumvent the nonhomogeneity of the Hartmann–Hahn condition at high rates of the spin rotor, a ^1^H ramp sequence was applied before transference to the ^13^C using a 2 ms contact time. The C chemical shifts were calibrated by utilizing the methylene signal of solid adamantane (29.5 ppm) as an external standard [42,47].

The NMR spectra were integrated to calculate the area of peaks appearing in the chosen regions. Seven chemical shift regions were identified for the following C types [48]: alkyl C (0~45 ppm), N-alkyl and methoxyl C (46~60 ppm), O-alkyl C (61~90 ppm), di-O-alkyl C (91~110 ppm), H- and C-substituted aromatic C (111~140 ppm), O-substituted aromatic C (141~160 ppm), and carbonyl C (161~190 ppm). The relative abundance (RA) of organic C was expressed as the area under these chemical shift regions relative to the total abundance, calculated with MestreNova software (Mestrelabs Research, Santiago de Compostela, Spain). The ratio of alkyl C to O-alkyl C (A/OA, Equation (1)) and the ratio of carbohydrate C to N-alkyl and methoxyl C (CC/MC, Equation (2)) were the proportion of labile to recalcitrant C. The ratio of N-alkyl and methoxyl C to O-substituted aromatic C (MC/PH, Equation (3)) was utilized to discriminate between signals due to lignin and other phenolic moieties. The ratios of aromatic C to O-alkyl C or carbonyl C were expressed as the proportion of recalcitrant C to labile organic C (AR/OA, Equation (4); AR/CA, Equation (5)). Aromaticity was used to calculate the ratio of aromatic C to total organic C (Equation (6)). Hydrophobicity was used to distinguish hydrophobic and hydrophilic functional groups (Equation (7)) [41,42,43,47,49,50]:

(1) $$\frac{A}{\mathrm{OA}}=\frac{\mathrm{alkyl}\;C}{O-\mathrm{alkyl}\;C}=\frac{\mathrm{RA}\left(0\;\sim \;45\right)}{\mathrm{RA}\left(61\;\sim \;110\right)}$$

(2) $$\frac{\mathrm{CC}}{\mathrm{MC}}=\frac{\mathrm{Carbohydarate}\;C}{\mathrm{Methoxyl}\;C}=\frac{\mathrm{RA}\left(61\;\sim \;90\right)}{\mathrm{RA}\left(46\;\sim \;60\right)}$$

(3) $$\frac{\mathrm{MC}}{\mathrm{PH}}=\frac{\mathrm{Methoxyl}\;C}{\mathrm{Phenol}\;C}=\frac{\mathrm{RA}\left(46\;\sim \;60\right)}{\mathrm{RA}\left(141\;\sim \;160\right)}$$

(4) $$\frac{\mathrm{AR}}{\mathrm{OA}}=\frac{\mathrm{Aromatic}\;C}{O-\mathrm{alkyl}\;C}=\frac{\mathrm{RA}\left(111\;\sim \;160\right)}{\mathrm{RA}\left(61\;\sim \;110\right)}$$

(5) $$\frac{\mathrm{AR}}{\mathrm{CA}}=\frac{\mathrm{Aromatic}\;C}{\mathrm{Carbonyl}\;C}=\frac{\mathrm{RA}\left(111\;\sim \;160\right)}{\mathrm{RA}\left(161\;\sim \;190\right)}$$

(6) $$\mathrm{Aromaticity}=\frac{\mathrm{Aromatic}\;C}{\mathrm{Alkyl}\;C+N-\mathrm{alkyl}\;\&\;\mathrm{methoxyl}\;C+O-\mathrm{alkyl}\;C+\mathrm{Aromatic}\;C}=\frac{\mathrm{RA}\left(111\;\sim \;160\right)}{\mathrm{RA}\left(0\;\sim \;160\right)}$$

(7) $$\mathrm{Hydrophobicity}=\frac{\mathrm{Hydrophobic}\;C}{\mathrm{Hydrophilic}\;C}=\frac{\mathrm{RA}\left(0\;\sim \;45\right)+\mathrm{RA}\left(111\;\sim \;160\right)}{\mathrm{RA}\left(61\;\sim \;110\right)+\mathrm{RA}\left(161\;\sim \;190\right)}$$

### 2.4. Enzyme Activity Assays

The following extracellular enzyme activities involved in C, nitrogen, and phosphorus were determined spectrophotometrically with minimal modification: cellobiohydrolase (CBH1), peroxidase (PERO), phenol oxidase (PHO), nitrate reductase (NR), and acid phosphatase (ACP). The methods of microbial enzymatic activity analysis followed the previous work [51]. We assayed microbial enzymatic activities in the surface of decomposing leaf litter on 288 days.

### 2.5. Statistical Analyses

The NMR datasets and mass loss were analyzed by three-way analysis of variance (ANOVA) to test significant differences and the interactions among various treatments (radiation waveband, water pulse, and decomposition time). Tukey’s honest significant difference (HSD) multicomparison test was used to examine significant differences between water treatments and among waveband treatments (*p* < 0.05). The NMR datasets were used to explain the variation in mass loss by applying random forest (RF) regression. The significance of each predictor on the response variable was assessed by the “rfPermute” package. Redundancy analysis (RDA) was used to visualize and quantify the magnitude of correlations between the NMR dataset and the environmental variables (radiation, water pulse, and extracellular enzyme activity). The permutation test (PT) was used to test the significance of the RDA model and each RDA axis. Variation partitioning analysis (VPA) was used to study the relative contribution of environmental variables to the variation in the NMR datasets. All statistical analyses and plots were performed by the computing environment R 4.1.1 (R Development Core Team, 2021) and the following packages: randomForest, rfPermute, vegan, and ggplot2.

## 3. Results

### 3.1. Changes in Litter Organic C under Exposure to Radiation Interacting with Water Pulses

**Figure 1:**
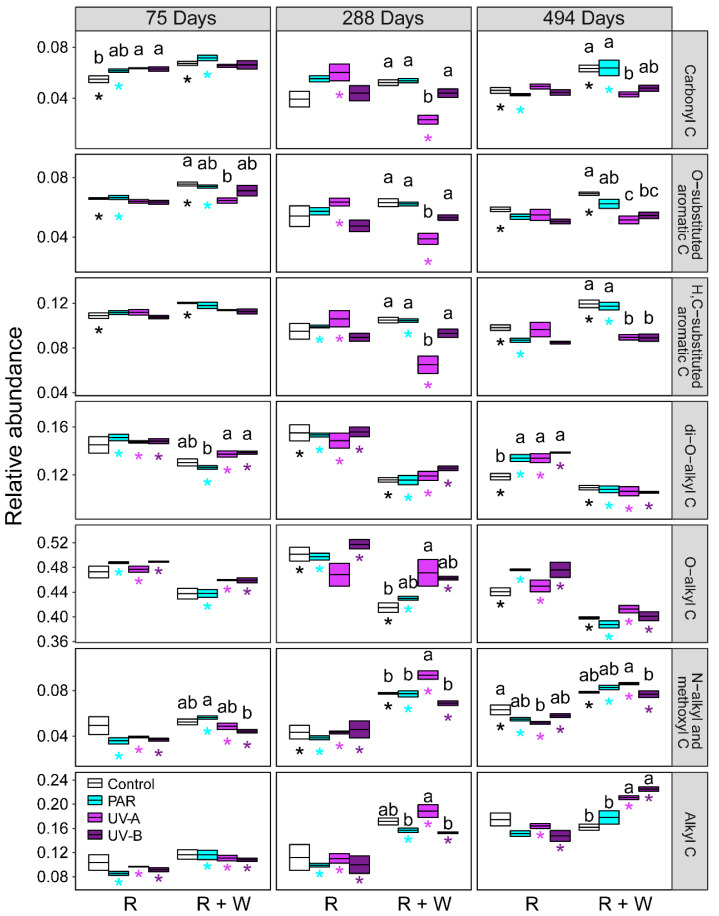
The relative abundance of chemical components of litter residues characterized by solid-state ^13^C cross-polarization magic angle spinning (CPMAS) nuclear magnetic resonance (NMR) spectra exposed to radiation without and with water pulses during different decomposition stages. Crossbars represent mean ± SE. Letters indicate significant differences among waveband treatments (control, PAR, UV-A, and UV-B) without or with water pulse treatments (R, radiation; R + W, radiation and water pulse) during every decomposition stage. Asterisks represent significant differences between radiation alone and the combination of radiation and water pulses in each waveband treatment during every decomposition stage (mean ± SE, *n* = 3, Tukey’s HSD test, *p* < 0.05).

Under radiation treatment (R), PAR and UV radiation had no effect on most organic C, compared to that of the blank (no radiation and no water) treatment, while UV-A radiation reduced the relative abundance of N-alkyl and methoxyl C (46~60 ppm) after 494 days of decomposition (*p* < 0.05, Figure 1 and Table S1). In the interaction between radiation and water pulses (R + W), compared to that of the blank treatment, the relative abundance of O-alkyl C (61~90 ppm) and di-O-alkyl C (91~110 ppm) was reduced in not only water pulses but also in the combined interaction between radiation (PAR and UV) and water pulses (R + W) during different decomposition stages (*p* < 0.05, Figure 1). In contrast, compared to water treatment alone, the relative abundance of H- and C-substituted aromatic C (111~140 ppm) and O-substituted aromatic C (141~160 ppm) was lower under UV radiation with water pulses (R + W) than under water pulses alone at the medium and last decomposition stages (*p* < 0.05, Figure 1). Carbonyl C (160~190 ppm) exhibited similar to that of decomposition pattern to aromatic C in litter residues exposed to the combined interaction of radiation and water pulses (R + W) (Figure 1).

### 3.2. The Importance of Chemical Components as Predictors of Litter Mass Loss

Both radiation wavebands and water pulses had a significant role in promoting litter mass loss (Figure S3 and Table S2). However, radiation (R) promoted approximately 3% mass loss over one year (Figure S3). PAR radiation (R) increased mass loss after 164 and 494 days of photodegradation (*p* < 0.05, Figure S3a). UV-A radiation (R) increased mass loss during all decomposition stages except for the first 75 days (Figure S3).

In contrast, the magnitude of mass loss caused by water pulses, whether with radiation or not, was 10 times that induced by radiation only during each decomposition stage (R + W) (Figure S3). Radiation mediated by water pulses (R + W) did not increase mass loss compared to water pulses alone, and similar decomposition patterns were exhibited among radiation wavebands at each decomposition stage (Figure S3). Mass loss of leaf litter exposed to water pulses was equal to that under water pulses mediated by PAR radiation for each decomposition time during medium and last decomposition stages (288, 380, and 494 days) and was significantly higher than that under water pulse mediated by UV radiation (*p* < 0.05, Figure S3). Moreover, no significant difference in magnitude of mass loss whether water pulses interacted with UV-A or UV-B radiation (R + W) (Figure S3).

**Figure 2:**
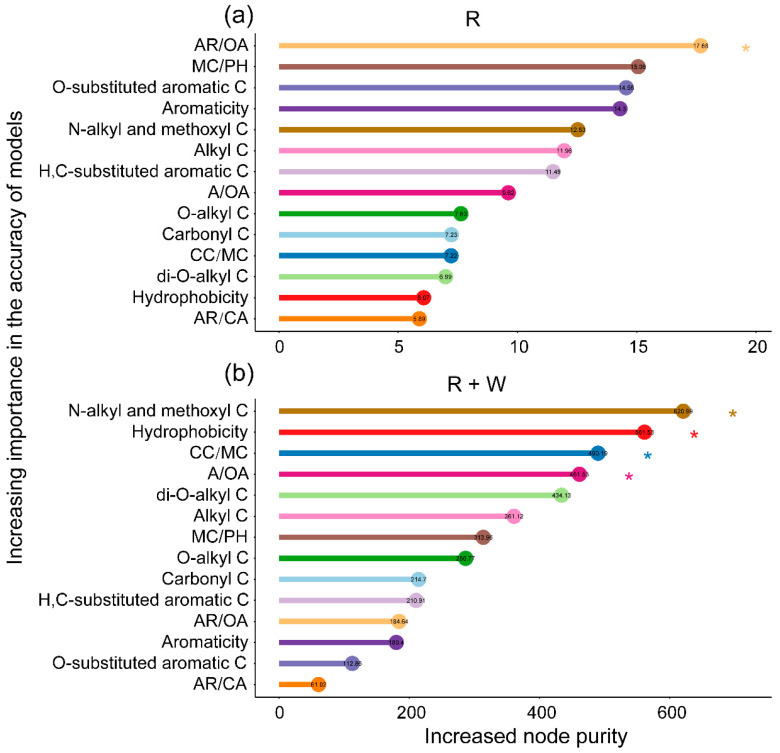
Decomposition indexes depending on the chemical composition of the ^13^C-CPMAS NMR dataset under exposure to radiation without and with water pulses were identified by applying random forest regression of their relative abundances from decomposing litter lasting 75, 288, and 494 days. (**a**) Plant litter exposed to radiation (R). (**b**) Plant litter exposed to the combined treatment between radiation and water pulses (R+W). Increased node purity was used to estimate the importance of these predictors, and higher values of increased node purity imply more important predictors (* *p* < 0.05).

The regression models explained 21.57% and 76.19% of the variation in mass loss related to plant-derived C under radiation without and with water pulses, respectively (Figure 2). The main decomposition indexes selected by random forest regression to explain the variation in mass loss were the ratio of aromatic C to O-alkyl C (AR/OA) in litter materials exposed to radiation alone (R) (Figure 2a), while the ratio of hydrophobic to hydrophilic C (hydrophobicity), the carbohydrate C to methoxyl C ratio (CC/MC), and the alkyl C to O-alkyl C ratio (A/OA) were the preferred indexes in litter materials exposed to radiation mediated by water pulses (R + W) (Figure 2b).

### 3.3. Relative Contributions of Abiotic and Biotic Drivers to Litter Decomposition

**Figure 3:**
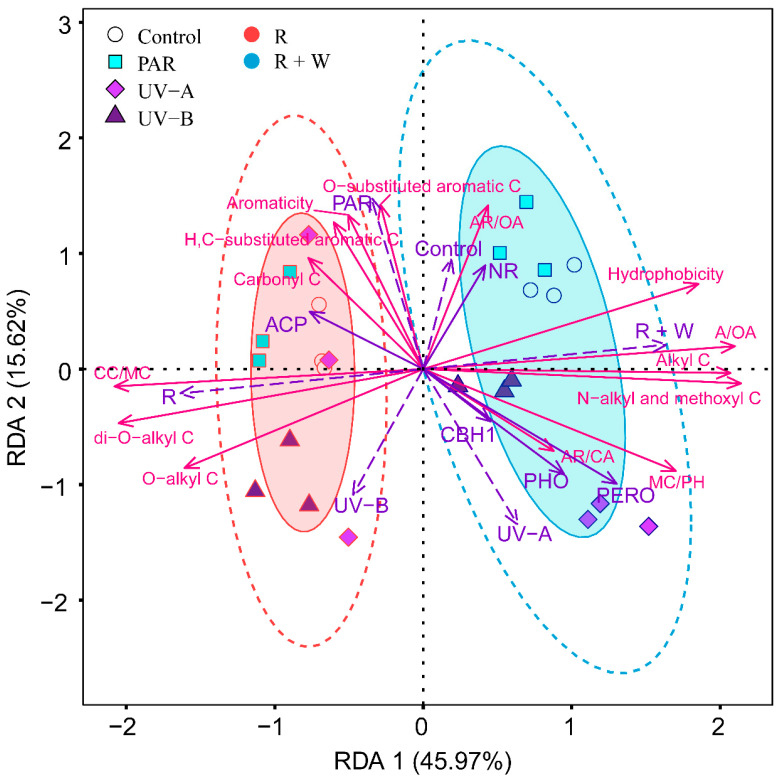
Redundancy analysis (RDA), a supervised learning approach, was applied to test the relationships between decomposition indexes depending on the ^13^C-CPMAS NMR dataset and environmental variables during litter decomposition that lasted 288 days. Litter materials exposed to radiation (waveband treatments: control, PAR, UV-A, and UV-B) without and with water pulse treatments (R, radiation; R + W, radiation and water pulse) in the microcosm experiment. Enzyme activities of the surface of leaf litter described by acid phosphatase (ACP), phenol oxidase (PHO), peroxidase (PERO), cellobiohydrolase (CBH1), and nitrate reductase (NR). Decomposition indexes were presented as vectors whose length and angle indicated the importance and direction of correlation with an ordination axis or another vector (magenta arrows). The environmental variables were presented as vectors whose length and angle indicated the importance and direction of correlation with an ordination axis or another vector (purple arrows). Shaded ellipses represent the 80% confidence interval, and dotted ellipse borders represent the 95% confidence interval. The positions of individual arrows along axes were set according to the loading scores, showing the relative importance along ordination axes.

The decomposition indexes characterized by solid-state ^13^C-CPMAS NMR spectra were influenced by extracellular enzyme activities of the litter surface, radiation wavebands, and water pulses. Water pulse was the dominant driver for decay indexes in axis 1 of the RDA (R and R + W). In contrast, UV and PAR radiation were the predictors in RDA ordination axis 2 (R). The decomposition indexes from the solid-state NMR dataset were distributed along gradients of water pulses in axis 1 of the RDA and scattered along radiation wavebands in axis 2 of the RDA (Figure 3).

**Figure 4:**
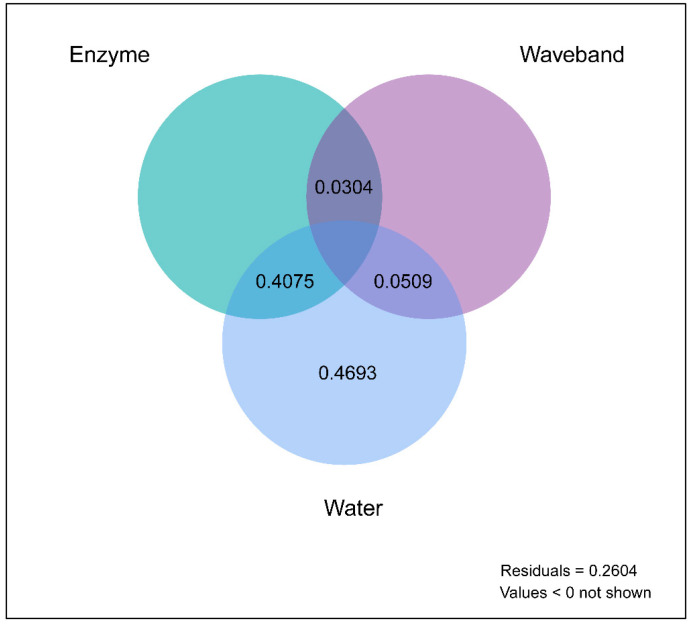
Variation partitioning analysis (VPA) was used to study the rates of relative contribution and interpretation of environmental variables to the variation in chemical composition characterized from the solid-state ^13^C-CPMAS NMR dataset during litter decomposition lasting 288 days. Radiation wavebands, water pulses, and enzyme activities were represented by waveband, water, and enzyme.

We performed variation partitioning analysis to evaluate the roles of radiation wavebands, water pulses, and enzyme activities in litter decomposition. Water pulses independently explained 46.93% of the variation in organic C. The interactive effect between water pulses and enzymatic activities explained 40.75% of the variation in organic C. The interactive effect between water pulses and radiation wavebands explained 5.09% of the variation in organic C. Alternatively, the combined effects of radiation wavebands and enzyme activities explained 3.04% of the variation in organic C (Figure 4).

## 4. Discussion

Litter chemistry, water availability, and microbial activities impact photodegradation magnitude in mesic ecosystems, thereby affecting the contribution of photodegradation to litter decomposition [12,15]. This study demonstrated that radiation (R) increased mass loss of plant litter, in contrast, the interaction between radiation and water pulses (R + W) did not increase mass loss compared to water pulses alone (Figure S3). There is increasing evidence that UV-B, UV-A and visible radiation promote litter C loss across different ecosystems [16,18,42,52]. In addition, our results are consistent with a recent study in which the effect of UV-B photolysis on litter decomposition was notable in a tropical rainforest, however, the mass loss of plant litter exposed to 90% blocked solar radiation was similar to that under full sunlight [12]. Photodegradation is a factor of litter decomposition in mesic ecosystems [13,14,17], however, the high amount of precipitation and microbial activity mask the photopriming effect of solar radiation on litter decomposition at the mass level [12,53].

The decomposition of litter organic C depends not only on the abundance and characteristics of chemical bonds but also on the energy absorbance of chemical bonds present within litter materials [54]. Our study supported the direct photolysis that lignin methoxyl C accounted for 7.46% of the total organic C that was photomineralized under UV-A radiation (Table S3). This result was consistent with the studies associated with photochemical decomposition at the molecular level (Figure 1). For instance, the relative abundance of syringyl units (S) characterized by solid-state ^13^C NMR in lignin decreased during leaf litter exposure to ambient solar radiation [55]. The relative abundances of lignin p-hydroxyphenyl units (H) and β-aryl ether linkages declined under UV photodegradation measured with solution-state 2D NMR [56]. The relative abundances of syringyl units (S) and guaiacyl units (G) decreased due to exposure to photodegradation analyzed by using a ^13^C-labeled tetramethylammonium hydroxide-thermochemolysis procedure in mesic deciduous forests [35]. Lignin is a random and complex phenolic polymer deposited in the secondary cell wall, and it can effectively absorb UV radiation [28,52]. Thus, the molecular structure of lignin will exert great effect on radiation (especially UV) mediated litter decomposition. The lignin polymer is composed of phenylpropanoid precursor units and the linkages between units. Lignin precursor units consist of syringyl-, guaiacyl-, and p-hydroxyphenyl-monolignols (S, G, and H) [56,57]. In addition, linkages include β-O-4 linkages between normal alcohol units, phenylcoumaran linkages between guaiacyl alcohol units, and resinol linkages that are often abundant in p-hydroxyphenyl lignin units [56,58]. Broadbands that appeared in the region (111~160 ppm) represented O-substituted aromatic C of lignin or other aromatic structures, and H- and C-substituted C of lignin monomers in guaiacyl and syringyl units [59,60]. The signal in the region (46~60 ppm) identified in the ^13^C NMR spectra is attributed to lignin methoxyl C [59,60]. All lignin monomers contribute to the region of methoxyl C, leading to this region being the most dominant lignin region [59].

O-alkyl C mainly represents carbohydrates, celluloses, and polysaccharides [45,54,55]. We did not observe the decrease of the relative abundance of O-alkyl C, which accounted for 58.64% of the total organic C under UV and PAR radiation exposure (Table S3). This result did not support the decomposition mechanism of indirect photolysis (Figure 1). However, cellulose O-alkyl C was lost when plant litter was exposed to the combined interaction among photodegradation, biodegradation, and water-pulse leaching. Soil microorganisms were reported to preferentially degrade labile cellulose and hemicellulose and to accumulate resistant lignin [30]. In addition, the leaching process caused by water pulses can lead to the loss of dissolved organic matter and cellulose [23,61]. Therefore, the decomposition pattern of O-alkyl C was similar to the traditional model of microbial decomposition.

However, compared to biodegradation alone, the photopriming effects of UV photodegradation on lignin aromatic C, rather than cellulose O-alkyl C appeared in the combined interaction between photodegradation and biodegradation (Figure 1). On the one hand, our result supported our second hypothesis that the photopriming effect of organic C would stimulate subsequent organic C decomposition mediated by water pulses. This was in line with previous studies showing that the photopriming effect increases extracellular enzyme activities by degrading lignin [62]. However, alternative studies showed that the photopriming effect was regulated by increasing accessibility to hemicellulose and cellulose protected by lignin [19,20,34]. The metabolic efficiency of microbial communities can vary with abiotic factors and substrate quality [63]. However, our results supported that photolysis facilitation and water availability increased the potential activities of peroxidase and phenol oxidase related to lignin degradation secreted by fungi (Figure S4). This indicated that the combined interaction between radiation and water pulses stimulated fungal activity with enhanced synthesis of oxidative enzymes. Long-term exposure could select for microbes that are more resistant to UV radiation or better able to use photo-oxidized products [64]. Baker and Allison (2015) supported that enzymatic activities (peroxidase and phenol oxidase) were positively associated with the change in lignin content during UV photodegradation. On the other hand, our study showed that UV photodegradation did not alter the chemical structure and relative abundance of cellulose O-alkyl C and then did not enhance the enzymatic activity (cellobiohydrolase) associated with cellulose degradation (Figure S4). UV radiation could suppress potential microbial activity by damaging microbial DNA and inhibiting microbial growth [65,66]. In addition, lignin protects cellulose and hemicellulose from microbial attack by physical and chemical mechanisms [58], which decreases accessibility to litter carbohydrates for microbial enzymes.

Solid-state NMR datasets provide more insight into the molecular-scale litter chemistry, which influences the variations in chemical components among plant litter types across terrestrial ecosystems [58]. Chemical compounds with various C functional groups show different sensitivities to photodegradation. Lignin with a more complex molecular structure appears to absorb greater radiation than less complex compounds, such as cellulose in the process of photodegradation [54]. The NMR dataset of organic C from plant litter was too complex to determine which organic C contributes to mass loss during litter decomposition. Hence, using chemical indexes to explain the variation in organic C during litter decomposition has the potential to reduce the complexity of the NMR dataset. Furthermore, decomposition indexes calculated by the ratios of different organic C were used to effectively analyze plant-derived C loss. Contrary to our third hypothesis, our study indicated that the robust decomposition indexes were AR/OA under radiation exposure. Alternatively, hydrophobicity, CC/MC, and A/OA were the robust decomposition indexes under radiation mediated by water pulses. The results were consistent with the pattern that cellulose is vulnerable to microbial decomposition, but lignin is resistant to microbial decomposition [28]. As di-O-alkyl C and O-alkyl C were degraded, aromatic C and alkyl C accumulated during the process of microbial decomposition, as shown by the solid-state ^13^C NMR spectra [45,46,51].

Previous research proved that the direct photodegradation process generates gas emissions [21,67]. Moreover, a study found that solar radiation accelerated leaching loss from plant litter [10]. Hence, we speculate that a part of the C loss caused by photodegradation is gas emissions via the direct photo-oxidation of organic C (lignin methoxyl C). With changes in the structures and abundances of lignin units and linkages, lignin may alter from complex macromolecules to small molecules or soluble organic C accelerates litter C loss through water-pulse leaching. At the same time, changes in lignin structures and abundances simulated fungi synthesized oxidized enzymes related to lignin decomposition, which further promoted the loss of litter C. The organic C loss caused by the direct and indirect effects of photodegradation on litter decomposition may be an unexplained part of the biochemical cycling model in humid ecosystems.

The dynamics of chemical components at the molecular level provided more comprehensive aspects to analyze plant-derived C loss and explain the consequences of the interaction between abiotic and biotic factors at the mass level during litter decomposition. ^13^C NMR is used to assess the structures and abundances of organic C in plant litter and to explore the potential decomposition mechanisms of photodegradation, which is hard to achieve by analyzing the changes in mass loss, lignin, cellulose, and hemicellulose of plant litter under exposure to photodegradation. Moreover, the new insight provided in our study is direct evidence of the photopriming effect of lignin aromatic C due to the photochemical mineralization of lignin methoxyl C.

The difference among syringyl, guaiacyl, and p-hydroxyphenyl monomer of lignin is the degree of methylation. Among them, there is no methoxyl group in the p-hydroxyphenyl monomer, while the guaiacyl monomer is in the 3-position oxygen methylation state of the benzene ring, and the syringyl monomer is in the 3-position and 5-position oxygen methylation state of the benzene ring. The methylation degree of the lignin monomer is related to the stability of the cell wall and degradation efficiency. In the future, we could use 2D-NMR to understand the structure and abundance of lignin units, and linkages are helpful to analyze the decomposition mechanism under the interaction of photodegradation and microbial decomposition. In addition, the traditional decomposition model based on biological metabolism underestimates the impact of the interaction of photodegradation with environmental factors and microbial communities on the decay rates of litter decomposition. Incorporating the turnover rates of different organic C pools (O-alkyl, alkyl, aromatic, and methoxyl C) and photodegradation as variables into the decomposition model can further improve the prediction power of the model.

## 5. Conclusions

**Figure 5:**
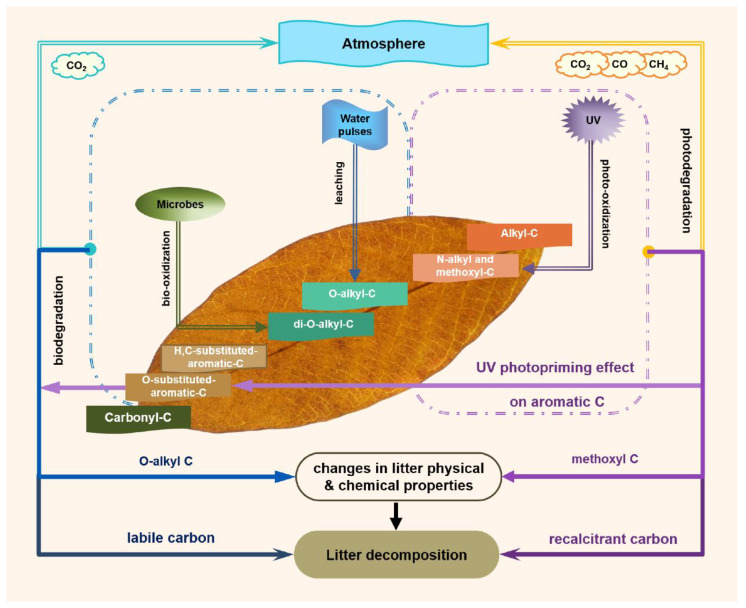
A decomposition paradigm of organic C in plant litter exposed to UV and PAR radiation and water pulses. Three independent pathways of litter decomposition: first, photochemical mineralization of methoxyl C exposed to UV radiation (purple dotted circle); second, the interaction between biodegradation and leaching of cellulose O-alkyl C in exposure to radiation interacting with water pulses (blue dotted circle); third, the photopriming effect of UV radiation on aromatic C in combined treatment of radiation and water pulses (purple dotted circle).

We presented three pathways of litter organic C decomposition that were driven by litter C characteristics during photodegradation (Figure 5). First, a photochemical path occurred when methoxyl C was photo-oxidized under exposure to UV radiation. Second, a combined path of biodegradation and water-pulse leaching occurred when O-alkyl C was degraded under water pulses. Third, the photopriming effects of UV radiation on aromatic C in radiation interacting with water pulses promoted subsequent microbial decomposition. The ratio of aromatic C to O-alkyl C (AR/OA) explained the variation in mass loss from plant litter exposed to photodegradation. In contrast, the proportion of labile to recalcitrant C (hydrophobicity, CC/MC, and A/OA) explained the variation in litter mass loss under the combined interactions among photodegradation, biodegradation, and water-pulse leaching. Our results revealed the decomposition mechanisms of organic C driven by abiotic and biotic factors, which is beneficial for accurately predicting the chemical changes of litter organic C and interpreting the nutrient cycle as well as energy flow among the atmosphere, organisms, and soil. Alternatively, we found that UV radiation had a photolysis effect on lignin methoxyl C and a photopriming effect on lignin aromatic C, indicating that the photo-oxidized compounds might be an additional C pool that affects the stability of the soil C pool by regulating the degradation of lignin methoxyl and aromatic C.

## Data Availability

Not applicable.

## Supplementary Materials

The following supporting information can be downloaded at: https://www.mdpi.com/article/10.3390/jof8090900/s1, Figure S1: Radiation wavebands and water pulses were controlled in a microcosm experiment of litter decomposition; Figure S2: Solid-state ^13^C cross-polarization magic angle spinning (CPMAS) nuclear magnetic resonance (NMR) spectra for initial chemical composition from leaf litter of *Lindera glauca*; Figure S3: The mass loss of leaf litter exposed to radiation mediated without and with water pulses during different decomposition stages in a microcosm experiment of litter decomposition; Figure S4: Microbial enzymatic activities in the surface of decomposing leaf litter on 288 days; Table S1: Chemical composition characterized by solid-state ^13^C-CPMAS NMR spectra analyzed by three-way analysis of variance (ANOVA); Table S2: The mass loss analyzed by three-way analysis of variance (ANOVA); Table S3: Solid-state ^13^C-CPMAS NMR spectra for initial chemical composition from leaf litter of *Lindera glauca*.
