# Supplementary material for: The Photodegradation of Lignin Methoxyl C Promotes Fungal Decomposition of Lignin Aromatic C Measured with 13C-CPMAS NMR
Source: J Fungi (Basel). 2022 Aug 24;8(9):900. doi: 10.3390/jof8090900 (PMC9504352; doi:10.3390/jof8090900)
Supplement: Supplementary file 1 [file jof-08-00900-s001.zip › jof-1859321-supplementary.pdf]

Supporting Information

Article title: **The photodegradation of lignin methoxyl C promotes fungal decomposition of lignin aromatic C measured with ^{13}C-CPMAS NMR**

Bei Yao[1], Xiaoyi Zeng[1], Lu Pang[1], Xiangshi Kong[1], Kai Tian[1], Yanli Ji[1], Shucun Sun[1] and Xingjun Tian[1, 2*]

[1]School of Life Sciences, Nanjing University, Nanjing 210023, China

[2]College of Eco-Environmental Engineering, Qinghai University, Xining, Qinghai, 810016, China

*Author of correspondence: Xingjun Tian

Tel: +86 13851857867

E-mail: tianxj@nju.edu.cn

ORCID

Xingjun Tian: https://orcid.org/0000-0002-0251-2582

Bei Yao: https://orcid.org/0000-0001-6434-1806

Xiangshi kong: https://orcid.org/0000-0003-3541-5663

The following Supporting Information is available for this article:

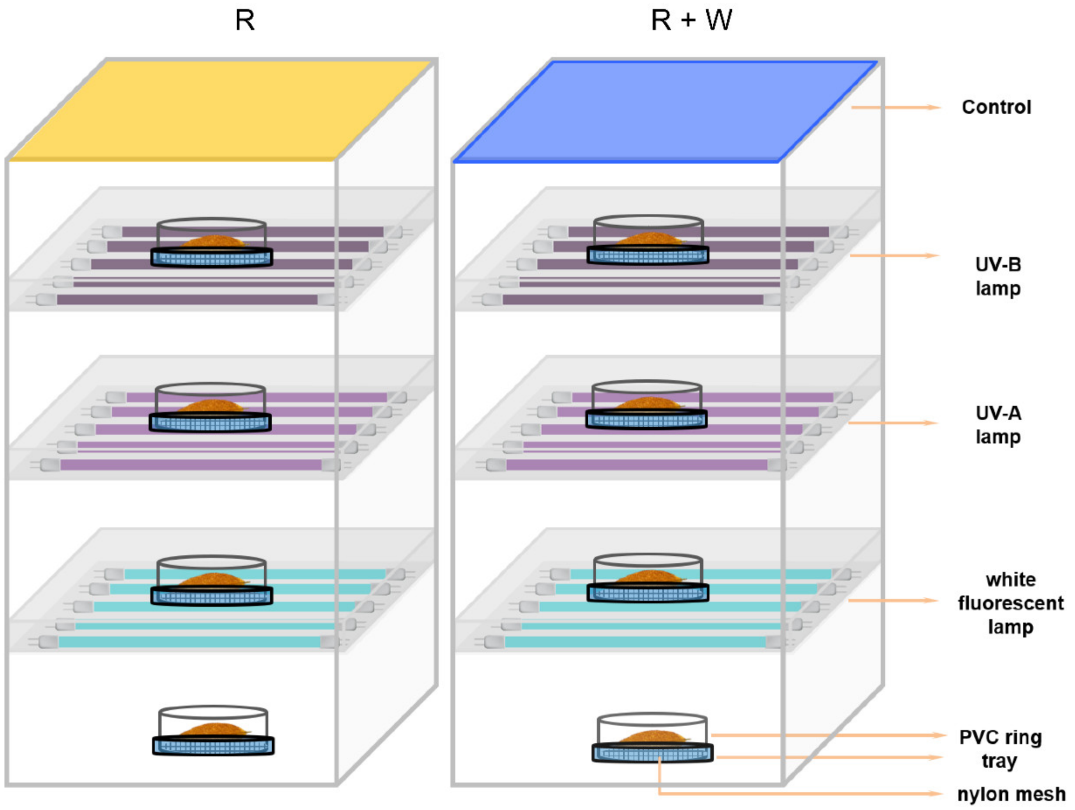

Figure S1 Radiation wavebands and water pulses were controlled in a microcosm experiment of litter decomposition. Litter materials were exposed to different types of fluorescent lamps (control, PAR, UV-A and UV-B) without and with water pulse treatments (R, radiation; R+W, radiation and water pulses) during each decomposition time.

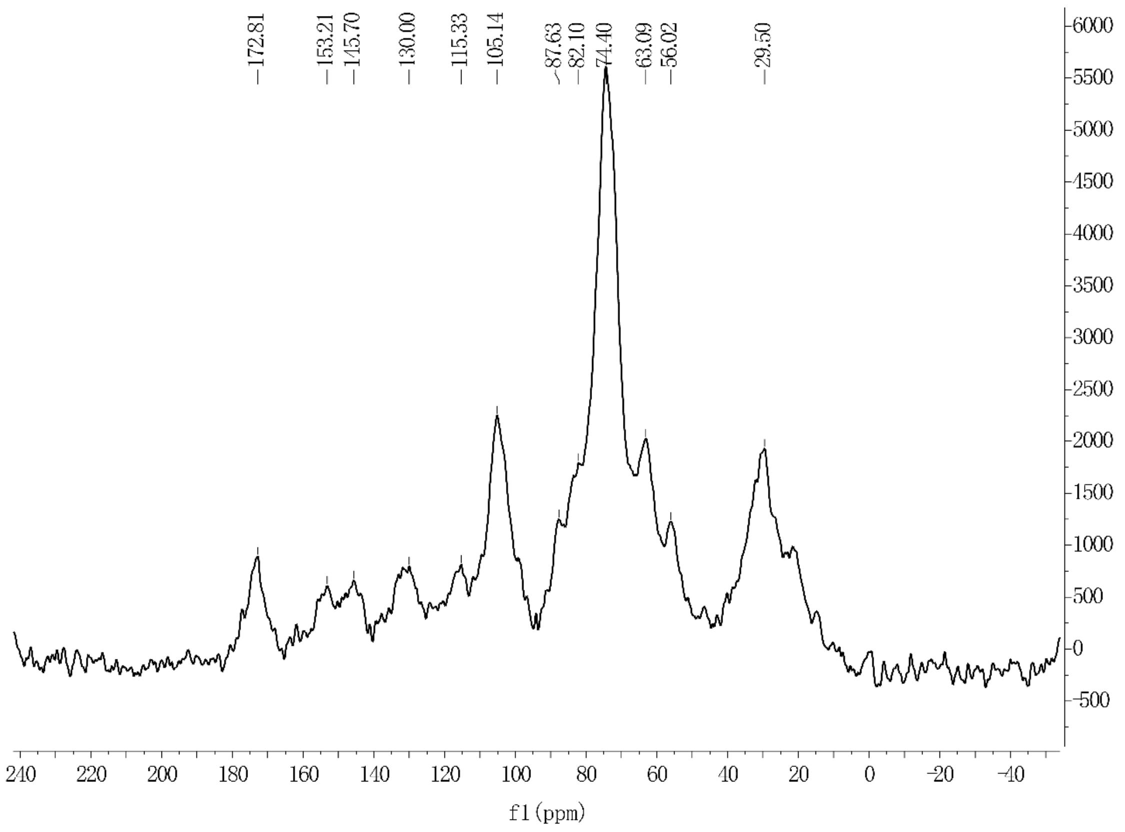

Figure S2 Solid-state ^{13}C cross-polarization magic angle spinning (CPMAS) nuclear magnetic resonance (NMR) spectra for initial chemical composition from leaf litter of *Lindera glauca*.

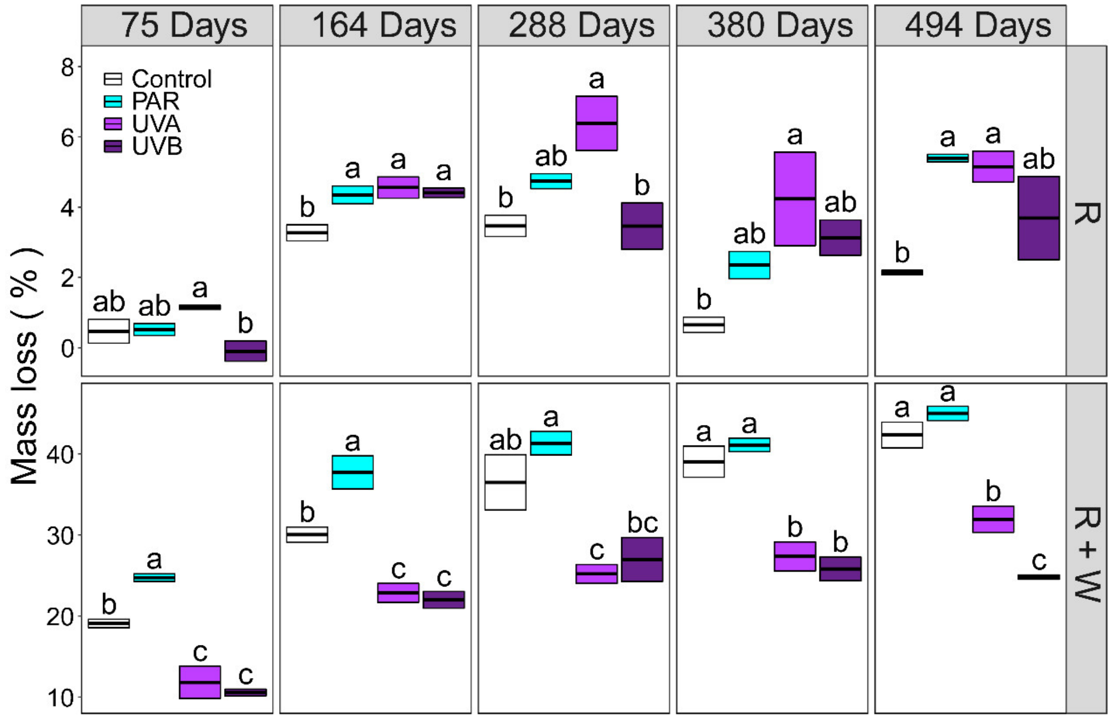

Figure S3 The mass loss of leaf litter exposed to radiation mediated without and with water pulses during different decomposition stages in a microcosm experiment of litter decomposition. Crossbars represent mean ± SE. Letters indicate significant differences among waveband treatments (control, PAR, UV-A and UV-B) without or with water pulse treatments (R, radiation; R+W, radiation and water pulses) during every decomposition stage (mean ± SE, n = 3, Tukey's HSD test, $P < 0.05$).

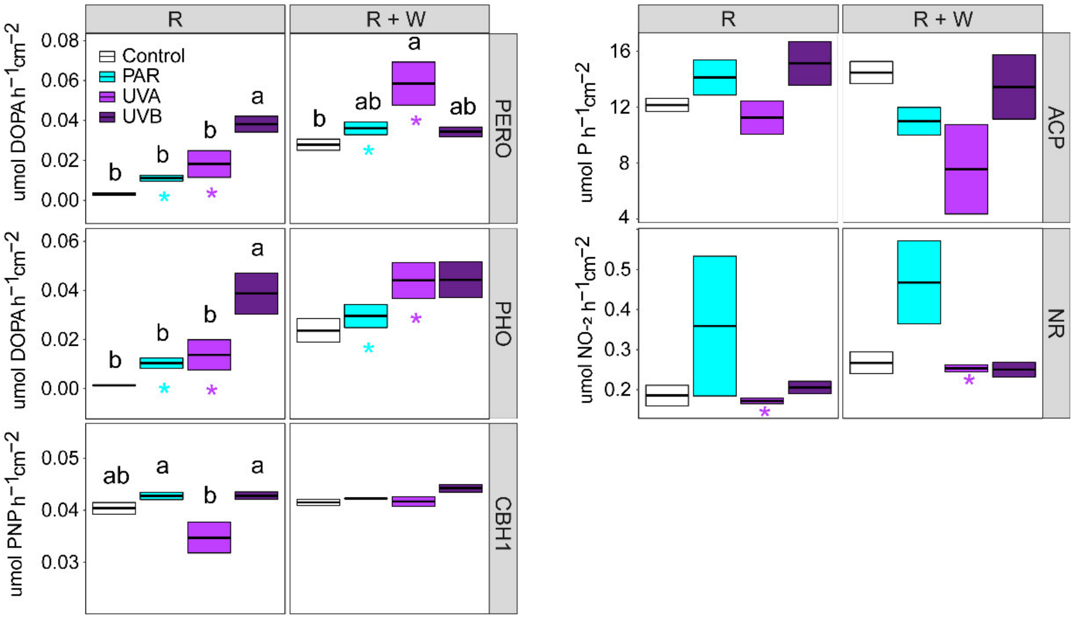

Figure S4 Microbial enzymatic activities in the surface of decomposing leaf litter on 288 days. Crossbars represent mean ± SE. Letters indicate significant differences among waveband treatments (control, PAR, UV-A and UV-B) without or with water pulse treatments (R, radiation; R+W, radiation and water pulses). Asterisks represent significant differences between radiation alone and the combination of radiation and water pulses in each waveband treatment (mean ± SE, n = 3, Tukey's HSD test, P < 0.05). PERO, peroxidase; PHO, phenol oxidase; CBH1, cellobiohydrolase; ACP, acid phosphatase; and NR, nitrate reductase.

Table S1 Chemical composition characterized by solid-state ^{13}C-CPMAS NMR spectra analyzed by three-way analysis of variance (ANOVA).

Factor	O-substituted aromatic C			H, C-substituted aromatic C			di-O-alkyl C			O-alkyl C		
	Df	F	*P*	Df	F	*P*	Df	F	*P*	Df	F	*P*
Waveband	3	14.64	**< 0.0001 *****	3	16.15	**< 0.0001 *****	3	3.60	**0.02 ***	3	7.67	**0.0003 *****
Water	1	9.42	**0.0035 ****	1	8.25	**0.006 ****	1	312.33	**< 0.0001 *****	1	193.87	**< 0.0001 *****
Time	2	57.11	**< 0.0001 *****	2	57.79	**< 0.0001 *****	2	88.54	**< 0.0001 *****	2	52.19	**< 0.0001 *****
Waveband × Water	3	15.25	**< 0.0001 *****	3	21.67	**< 0.0001 *****	3	1.84	0.1525	3	9.86	**< 0.0001 *****
Waveband × Time	6	1.27	0.2897	6	3.42	**0.0068 ****	6	0.90	0.5006	6	0.57	0.7535
Water × Time	2	4.65	**0.0142 ***	2	12.08	**0.0001 *****	2	16.33	**< 0.0001 *****	2	5.44	**0.0074 ****
Waveband × Water × Time	6	2.96	**0.0153 ***	6	5.28	**0.0003 *****	6	3.06	**0.0128 ***	6	2.90	**0.0171 ***

Factor	Alkyl C			N-alkyl and methoxyl C			Carbonyl C		
	Df	F	*P*	Df	F	*P*	Df	F	*P*
Waveband	3	3.69	**0.0181 ***	3	3.93	**0.0138 ***	3	6.25	**0.0011 ****
Water	1	128.59	**< 0.0001 *****	1	318.92	**< 0.0001 *****	1	5.47	**0.0235 ***
Time	2	155.45	**< 0.0001 *****	2	109.85	**< 0.0001 *****	2	66.10	**< 0.0001 *****
Waveband × Water	3	3.77	**0.0165 ***	3	8.37	**0.0001 *****	3	21.47	**< 0.0001 *****
Waveband × Time	6	2.28	0.0509	6	2.43	**0.0393 ***	6	2.34	**0.0458 ***
Water × Time	2	13.97	**< 0.0001 *****	2	33.59	**< 0.0001 *****	2	12.94	**< 0.0001 *****
Waveband × Water × Time	6	4.03	**0.0024 ****	6	1.90	0.0993	6	4.44	**0.0012 ****

Degree of freedom, F-value and P statistic are represented by Df, F and P respectively in the table. The decomposition time, water pulse, and radiation waveband are represented by time, water, and waveband, respectively. Significance levels are as follows: *** 0.001 ** 0.01 * 0.05.

Table S2 The mass loss analyzed by three-way analysis of variance (ANOVA).

Factor	DF	F	*P*
Waveband	3	98.58	**< 0.0001 *****
Water	1	4914.54	**< 0.0001 *****
Time	4	118.10	**< 0.0001 *****
Waveband × Water	3	114.21	**< 0.0001 *****
Waveband × Time	12	1.09	0.3799
Water × Time	4	59.13	**< 0.0001 *****
Waveband × Water × Time	12	2.01	**0.0337***

Degree of freedom, F-value and P statistic are represented by Df, F and P respectively in the table. The decomposition time, water pulse, and radiation waveband are represented by time, water, and waveband, respectively. Significance levels are as follows: *** 0.001 ** 0.01 * 0.05.

Table S3 Solid-state ^{13}C-CPMAS NMR spectra for initial chemical composition from leaf litter of *Lindera glauca*.

Carbonyl C	O-substituted aromatic C	H, C-substituted aromatic C	di-O-alkyl C	O-alkyl C	N-alkyl and methoxyl C	Alkyl C
0.0227±0.0037	0.0442±0.0042	0.0754±0.0123	0.1211±0.0044	0.4653±0.0067	0.0746±0.0044	0.1968±0.0172

mean ± SE, n = 3
